# Biomarkers for Early Detection of Cisplatin-Induced Nephrotoxicity

**DOI:** 10.3390/life15091432

**Published:** 2025-09-12

**Authors:** Nikolay Dimov, Antoniya Yaneva, Evelina Valcheva, Gabriela Raycheva, Veselin Popov, Raya Delipavlova, Dimitar Nikolov, Zhanet Grudeva-Popova

**Affiliations:** 1 Nephrology Section, Second Department of Internal Diseases, Medical University of Plovdiv, 15A Vasil Aprilov Blvd., 4002 Plovdiv, Bulgaria; 2 Clinic of Nephrology, University Multiprofile Hospital for Active Treatment “Sv. Georgi”—Plovdiv, 15A Vasil Aprilov Blvd., 4002 Plovdiv, Bulgaria; evelinavalcheva98@gmail.com; 3 Department of Medical Informatics, Biostatistics and E-learning, Medical University of Plovdiv, 15A Vasil Aprilov Blvd., 4002 Plovdiv, Bulgaria; yaneva.antonya@gmail.com; 4 Department of Clinical Oncology, Medical University of Plovdiv, 15A Vasil Aprilov Blvd., 4002 Plovdiv, Bulgaria; dr.graycheva@gmail.com (G.R.); dr.v_popov@yahoo.com (V.P.);; 5 Clinic of Medical Oncology, University Multiprofile Hospital for Active Treatment “Sv. Georgi”—Plovdiv, 15A Vasil Aprilov Blvd., 4002 Plovdiv, Bulgaria; 6 Clinic of Radiation Oncology, University Multiprofile Hospital for Active Treatment “Sv. Georgi”—Plovdiv, 15A Vasil Aprilov Blvd., 4002 Plovdiv, Bulgaria; 7 Department of Clinical Laboratory, Medical University of Plovdiv, 15A Vasil Aprilov Blvd., 4002 Plovdiv, Bulgaria; raya.delipavlova@phd.mu-plovdiv.bg; 8 Clinic of Clinical Laboratory, University Multiprofile Hospital for Active Treatment “Sv. Georgi”—Plovdiv, 15A Vasil Aprilov Blvd., 4002 Plovdiv, Bulgaria; 9 Clinic of Clinical Hematology, University Multiprofile Hospital for Active Treatment “Sv. Georgi”—Plovdiv, 15A Vasil Aprilov Blvd., 4002 Plovdiv, Bulgaria

**Keywords:** uKIM-1, uClusterin, uNephrin, cystatin C, nephrotoxicity, platinum-based therapy, eGFR decline

## Abstract

Nephrotoxicity is a common complication during antineoplastic therapy, particularly when platinum-based medications are used. Early detection of this condition is crucial for improving risk stratification and management, thereby enhancing decision-making in kidney disease treatment. However, traditional biomarkers for renal assessment lack sensitivity in identifying early or subclinical damage, underscoring the need for novel and more precise markers. This study aimed to investigate the effectiveness of urinary KIM-1, clusterin, nephrin, and serum cystatin C in detecting nephrotoxicity associated with platinum-based therapies. A total of 43 patients with different oncological diseases participated in the prospective study, divided into two groups based on the nephrotoxic potential of the administered drugs: patients treated with cisplatin (high-risk group for nephrotoxicity) and patients treated with oxaliplatin/carboplatin (low-to-moderate risk group for nephrotoxicity). The results showed that nephrotoxicity, determined as a decrease in eGFR of >10 mL/min/1.73 m^2^ at the sixth month after initiation of platinum-based therapy, occurred in 54.3% of cases, with 80% of these attributable to cisplatin-based therapy. Conventional renal biomarkers, such as the serum creatinine and urine albumin-creatinine ratio, have shown controversial results in the course of the study. In contrast, the patients treated with cisplatin, as well as those who developed nephrotoxicity, showed significant increases in the mean values of cystatin C (*p* < 0.001, respectively, *p* < 0.001), urinary KIM-1 (*p* = 0.005, respectively, *p* = 0.002), and urinary clusterin (*p* = 0.001, respectively, *p* = 0.001). Among the group with a low to moderate risk of nephrotoxicity including those treated with oxaliplatin/carboplatin, no statistically significant changes over time were observed in any of the biomarkers. These findings suggest that the aforementioned biomarkers can be used for the early detection of cisplatin-induced eGFR decline.

## 1. Introduction

Cancer presents a significant global health challenge. By 2050, the number of cancer cases worldwide is projected to reach 35.3 million, marking a 76.6% rise compared to the estimated 20 million cases in 2022 [1]. As a consequence, from 2017 to 2022, the US Food and Drug Administration (FDA) authorised 161 new cancer therapies for solid tumours, significantly exceeding the 71 approvals recorded between 2002 and 2014 [2]. Additionally, annual antineoplastic prescriptions have increased, with those for colorectal cancer rising 80.4% from 2015 to 2019, and those for lung cancer increasing 85.6% in 2020 [3,4]. Unfortunately, anticancer drugs are associated with a wide range of toxic effects. Nephrotoxicity is a common side effect during cancer treatment, with 80.1% of patients receiving potentially nephrotoxic drugs as they undergo cancer therapy [5].

Platinum-based drugs are among the foundational chemotherapy agents utilised in treating oncology patients. Main representatives of this group are cisplatin, carboplatin, oxaliplatin, and nedaplatin [6,7]. When administering platinum-based drugs, a cancer patient might experience a combination of approximately 40 different specific side effects [8]. Nephrotoxicity is one of the most frequent and significant among them [8].

However, the occurrence of platinum-induced nephrotoxicity manifests to varying degrees depending on the specific medication, being especially evident in the case of cisplatin [9,10].

In general, drug-induced nephrotoxicity varies from an acute or chronic reduced glomerular filtration rate (GFR) to nephrotic syndrome and electrolyte disorders that are related, respectively, to glomerular and tubular damage [11]. Antineoplastic drugs (including platinum-based therapy) not only may affect the nephron in multiple compartments but are also used in various combinations and regimens, which further complicates the precise identification of the drug or drugs responsible for the renal damage. The appearance of renal toxicity can potentially lead to the need for dose adjustment or cessation of therapy. This necessitates the prompt detection of potential early kidney damage. Current laboratory tests are unable to predict patients at risk or detect the early stages of the disease. The timely diagnosis of early kidney damage requires the application of novel biomarkers, which are more sensitive and highly specific and can also provide more precise information about the exact location of renal injury.

Promising biomarkers in this context are urinary KIM-1 (uKIM-1), urinary Clusterin (uClusterin), urinary Nephrin (uNephrin), and cystatin C (Cys C), which have been reported in multiple studies describing drug-induced nephrotoxicity [12,13,14].

KIM-1 is a proximal tubule apical transmembrane protein that is expressed at low concentrations in normal kidneys, but its level dramatically increases in dedifferentiated proximal tubular cells in the presence of ischaemic or nephrotoxic acute kidney injury [15,16,17]. uClusterin is a glycoprotein used to detect proximal tubular injury, with an ability to mirror damage across various nephron territories, including the distal tubule [16,18]. Cystatin C is a low-molecular-weight protein expressed ubiquitously in all nucleated cells. It is primarily eliminated by glomerular filtration, and therefore, the serum cystatin C concentration has been used as a novel endogenous marker of the GFR [16,19]. Nephrin is a 180 kD transmembrane protein expressed in podocytes and localised in the slit diaphragm of these cells, where it performs structural and signalling functions [20]. Urinary nephrin may serve as a promising marker for the detection of early glomerular injury [21]. Being commonly used in oncology patients, platinum-based regimens demand close monitoring due to the high risk of side effects, including the occurrence of nephrotoxicity, the diagnosis and treatment of which require a comprehensive approach, including the use of novel biomarkers.

## 2. Aim

The aim of this pilot study was to assess the role of uKIM-1, uClusterin, uNephrin, and serum Cystatin C biomarkers in detecting early signs of renal injury associated with platinum-induced nephrotoxicity.

## 3. Materials and Methods

### 3.1. Study Design and Participants

This prospective two-center observational pilot study was conducted at the University Hospital “Sveti Georgi”—Plovdiv and “Complex Oncological Center”—Plovdiv, enrolling patients undergoing platinum-based chemotherapy together with healthy control group. Patients were stratified into two groups based on their risk of developing nephrotoxicity: a high-risk group and a low-to-moderate-risk group [9,10].

- High-risk group for nephrotoxicity: patients treated with cisplatin;
- Low-to-moderate risk group for nephrotoxicity: patients treated with oxaliplatin/carboplatin.

### 3.2. Eligibility Criteria

#### 3.2.1. Inclusion Criteria

Patients who were eligible included those over age 18 years and who had provided signed informed consent; patients with histologically confirmed oncological disease, referred for a first course of systemic antineoplastic treatment; patients without evidence of kidney impairment at the time of the study; and patients receiving antineoplastic drug therapy based on platinum-based conventional chemotherapy.

#### 3.2.2. Exclusion Criteria

Ineligible participants included those without signed informed consent to undergo the study; patients under 18 years of age; patients with underlying kidney disease at the time of the study; patients with histologically confirmed malignant neoplasm of the kidney; patients prescribed an antineoplastic drug regimen that did not include a platinum-based drug; and patients prescribed an antineoplastic drug regimen in combination with immunotherapy and/or target therapy.

### 3.3. Sample Collection and Follow-Up

Venous blood and urine samples were taken under standard conditions once from participants in the control group (t0) and four times from each patient (t0–t3), as follows: on the day of hospitalisation of each patient (t0 sample); one day after the first cycle of antineoplastic therapy (t1 sample); one day after the second cycle of antineoplastic therapy (t2 sample); and one day after the third cycle of antineoplastic therapy (t3 sample). Antitumor drug regiments were administrated according to European Society For Medical Oncology guidelines (in cycles of three to four weeks).

The blood tests included full blood count, lipid profile, blood glucose, electrolytes, serum creatinine, urea, uric acid, cystatin C, and estimated glomerular filtration rate (eGFR) calculated using the CKD-EPI Creatinine Equation (2021).

The urine tests included standard urine examination, urine albumin-creatinine ratio (uACR), uClusterin, uNephrin, and uKIM-1.

For the analysis of uClusterin, uNephrin, and uKIM-1, the respective research use only kits were used:

- Human Nephrin ELISA kit, 96 tests, Mybiosource, catalog number: MBS265927;
- Human Kim-1 ELISA kit, 96 tests, Mybiosource, catalog number: MBS454373;
- Clusterin Human ELISA, 96 tests, Biovendor, catalog number: RD194034200R.

The concentrations of these markers were measured using the ELISA method according to the kit manufacturer’s instructions.

### 3.4. Definitions and Terminology; Outcome Assessment

Renal function was assessed using conventional and novel biomarkers.

Conventional markers: serum creatinine, eGFR (CKD-EPI Creatinine Equation—2021), and u-ACR.

Novel biomarkers: cystatin C, uClusterin, uNephrin, and uKIM-1.

For patients without evidence of kidney impairment at the time of examination, we define these patients as those for whom, based on medical history, physical status, imaging methods, and conventional laboratory methods for assessing kidney function, no evidence of specific kidney disease is found and/or who do not meet the Kidney Disease: Improving Global Outcomes (KDIGO) criteria for defining kidney disease, including CKD, AKI, etc.

For the purposes of the present study, nephrotoxicity was evaluated and monitored solely through changes in eGFR. We defined nephrotoxicity as a decrease in eGFR >10 mL/min/m^2^ at the sixth month following treatment initiation.

Patients were categorised into two groups based on renal outcome:

- eGFR decline >10 mL/min/1.73 m^2^—patients with developed nephrotoxicity;
- No eGFR decline or eGFR decline ≤10 mL/min/1.73 m^2^—patients with stable kidney function.

### 3.5. Statistical Analysis

Descriptive statistics were used to summarize demographic and clinical characteristics. Continuous variables were expressed as mean ± standard deviation or median (interquartile range), and categorical variables as counts and percentages. 

Comparisons between groups were performed using the Mann–Whitney U test for continuous variables, and the chi-square or Fisher’s exact test for categorical variables. The Kruskal–Wallis H test was applied for comparing conventional and novel biomarkers at baseline across the three groups, followed by post hoc pairwise comparisons with Bonferroni adjustment where appropriate. Changes in biomarkers over time (t0, t1, t2, t3) were assessed using Friedman’s test for repeated measures. When significant, Bonferroni-adjusted Wilcoxon signed-rank tests were used for post hoc pairwise comparisons between time points. A two-tailed *p*-value < 0.05 was considered statistically significant. All analyses were conducted using IBM SPSS Statistics version 23.

## 4. Results

### 4.1. Participants Characteristics

The study included a total of 66 participants, comprising 43 patients undergoing platinum-based chemotherapy and 23 healthy controls. Among the patients, 55.8% (*n* = 24) were classified as high risk for nephrotoxicity development (Cisplatin group), while 44.2% (*n* = 19) were categorized as low to moderate risk (Carboplatin/Oxaliplatin group). Regarding chemotherapy regimens, 55.8% (*n* = 24) received cisplatin, 25.6% (*n* = 11) received oxaliplatin, and 18.6% (*n* = 8) were treated with carboplatin. The demographic and clinical characteristics of patients stratified into low-to-moderate-risk and high-risk groups for nephrotoxicity, along with those of the control group are summarized in Table 1.

A non-parametric Kruskal–Wallis H test revealed a statistically significant difference in age across groups, H(2) = 34.76, *p* < 0.001. Post hoc analysis of mean ranks showed that participants in the control group (mean rank = 14.48) were significantly younger than those in the Cisplatin (mean rank = 43.04) and Carboplatin/Oxaliplatin (mean rank = 44.47) groups. No statistically significant difference in age was observed between the two treatment groups.

A chi-square test revealed a significant association between group and sex, χ^2^(2, *n* = 66) = 11.78, *p* = 0.003, indicating that sex differed significantly across the Cisplatin, Carboplatin/Oxaliplatin, and control groups.

No significant differences were found between the Cisplatin and Carboplatin/Oxaliplatin groups in the clinical characteristics: hypertension (χ^2^(1) = 0.06, *p* = 0.811), anemia (χ^2^(1) = 0.06, *p* = 0.807), or diabetes (χ^2^(1) = 0.10, *p* = 0.757).

### 4.2. Tumor Localization and Chemotherapy Regimens

The most common malignancies were digestive/gastrointestinal followed by those of the genitourinary tract (Table 2).

Regarding the applied antineoplastic drug regimens, the most common combinations were as follows: for cisplatin-based therapy—Cisplatin/Gemcitabine—37.5% (*n* = 9), for carboplatin-based therapy—Carboplatin/Paclitaxel—36.9% (*n* = 7), and for oxaliplatin-based therapy—FOLFOX-4—26.3% (*n* = 5). A detailed description of all applied antineoplastic regimens is provided in the Supplementary Materials (Supplementary Tables S1 and S2).

### 4.3. Group Differences in Renal Biomarkers at Baseline

At baseline (t0), all patients in both the Cisplatin and Carboplatin/Oxaliplatin groups had creatinine and uACR values within the reference range. No significant differences were observed between the treatment groups in baseline eGFR (U = 218.00, *p* = 0.807). However, mean serum cystatin C exceeded the upper reference limit (Cisplatin Group 1.46 ± 0.60 mg/L, Carboplatin/Oxaliplatin Group 1.46 ± 0.63 mg/L), with 74.4% (*n* = 32) of patients demonstrating abnormally elevated levels (reference ranges 0.51–1.09 mg/L). Among the Cisplatin group, 75.0% (18/24) had abnormal values, compared to 73.7% (14/19) in the Carboplatin/Oxaliplatin group. The difference in proportions was not statistically significant, χ^2^(1, *n* = 43) = 0.01, *p* = 0.919 (Table 3).

A Kruskal–Wallis H test was conducted to compare baseline levels of uKIM-1, uNephrin, and uClusterin across the three groups. The results indicated a statistically significant difference in uKIM-1 levels, H(2) = 16.15, *p* < 0.001. At baseline, both the Cisplatin and Carboplatin/Oxaliplatin groups had higher uKIM-1 levels compared to the control group. The increase was significant for the Cisplatin group (U = 89.00, *Z* = −3.98, *p* < 0.001) and also for the Carboplatin/Oxaliplatin group (U = 129.00, *Z* = −2.26, *p* = 0.024). In contrast, no significant differences were observed in uNephrin levels between the Cisplatin and control groups (U = 254.00, *p* = 0.624), nor between the Carboplatin/Oxaliplatin and control groups (U = 200.00, *p* = 0.615). Similarly, uClusterin levels did not differ significantly between Cisplatin and control (U = 261.00, *p* = 0.749), or between Carboplatin/Oxaliplatin and control (U = 212.00, *p* = 0.869) (Table 3).

### 4.4. Renal Biomarker Dynamic Across Chemotherapy Timepoints (T0–T3)

#### 4.4.1. Within Group Analysis: Cisplatin Group

In Cisplatin Group, a progressive increase in serum creatinine was observed across time points (T0–T3). However, levels exceeded the reference range in only 4 individuals. Similarly, a progressive increase in uACR was observed across the time points (T0–T3). However, mean levels exceeded the reference range only in T3, where a significant increase from T0 to T3 (*Z* = −2.907, *p* = 0.004, adjusted *p* = 0.022) was noted. Descriptive statistics for serum creatinine and other renal biomarkers are presented in Table 4.

In the Cisplatin group (*n* = 24), repeated-measures analysis using the Friedman test revealed statistically significant changes over time in all four renal biomarkers. Serum Cystatin C levels changed significantly across the four time points, χ^2^(3) = 27.15, *p* < 0.001. Post hoc Wilcoxon signed-rank tests showed increases from baseline (T0) to T1 (*Z* = −2.91, *p* = 0.022), T2 (*Z* = −3.69, *p* = 0.001), and T3 (*Z* = −5.03, *p* < 0.001). Urinary KIM-1 also varied significantly over time, χ^2^(3) = 12.67, *p* = 0.005. Bonferroni-adjusted post hoc comparisons revealed a significant increase between T0 and T3 (*Z* = −2.96, *p* = 0.003, adjusted *p* = 0.018), while other pairwise differences were not significant after correction. uNephrin levels demonstrated significant time-dependent variation, χ^2^(3) = 14.19, *p* = 0.003. Significant increases were observed between T0 and T2 (*Z* = −3.41, *p* = 0.001, adjusted *p* = 0.004) and between T0 and T3 (*Z* = −2.74, *p* = 0.006, adjusted *p* = 0.037). The increase from T0 to T1 approached significance (*p* = 0.034) but did not remain significant after correction (adjusted *p* = 0.202). Finally, uClusterin levels changed significantly over time, χ^2^(3) = 15.67, *p* = 0.001. A significant increase was noted between T0 and T3 (*Z* = −3.80, *p* < 0.001, adjusted *p* = 0.001), with no other comparisons reaching statistical significance (Table 5).

In the Cisplatin group (*n* = 24), Friedman tests were performed to assess changes in fold change values for urinary renal biomarkers across chemotherapy cycles (T1–T3), (Table 6).

In the Cisplatin group, fold changes in cystatin C differed significantly across time points (T0–T3), χ^2^(2) = 7.58, *p* = 0.023. Post hoc comparisons revealed a significant increase from T0 to T3 (*Z* = −2.742, *p* = 0.006, adj. *p* = 0.018). The results indicated no statistically significant differences in fold change for uKIM-1 (χ^2^(2) = 1.23, *p* = 0.541) or uNephrin (χ^2^(2) = 2.49, *p* = 0.287). However, the fold change in uClusterin approached statistical significance (χ^2^(2) = 5.94, *p* = 0.051), suggesting a possible upward trend across treatment timepoints that may warrant further exploration in larger samples (Table 7).

#### 4.4.2. Within Group Analysis: Carboplatin/Oxaliplatin Group

In the Carboplatin/Oxaliplatin group, all 19 patients in the Carboplatin + Oxaliplatin group had serum creatinine levels within the reference range at all four measured time points (t0–t3), indicating no deviations in renal function based on this conventional marker (Table 8). Additionally, eGFR values did not differ significantly across time points (T0–T3), χ^2^(3) = 3.33, *p* = 0.344. In contrast, a significant difference was observed in uACR values over time, χ^2^(3, *n* = 19) = 16.07, *p* = 0.001, with post hoc comparisons revealing a significant increase from T0 to T3 (*Z* = −3.204, *p* = 0.001, adj. *p* = 0.008). To evaluate temporal changes in novel and conventional renal biomarkers, Friedman tests were conducted for each marker across four time points. The results did not show statistically significant differences over time for any of the biomarkers Cystatin C: χ^2^(3) = 1.80, *p* = 0.615, uKIM-1: χ^2^(3) = 2.90, *p* = 0.407, uNephrin: χ^2^(3) = 1.97, *p* = 0.580, uClusterin: χ^2^(3) = 2.10, *p* = 0.552 (Table 9).

In the Carboplatin/Oxaliplatin (*n* = 19), Fold Change in Renal Biomarkers over time is presented in Table 10.

In the Carboplatin/Oxaliplatin group (*n* = 19), fold change values for serum cystatin C (χ^2^(2) = 0.74, *p* = 0.692), uKIM-1 (χ^2^(2) = 0.54, *p* = 0.765), uClusterin (χ^2^(2) = 3.12, *p* = 0.210), and uNephrin (χ^2^(2) = 0.83, *p* = 0.662) did not differ significantly across the three measured time points. These results suggest stable biomarker responses in this group over the treatment course (Table 11).

#### 4.4.3. Between-Group Comparisons

At baseline (T0), serum creatinine levels were within the reference range for all patients in both the Cisplatin and Carboplatin/Oxaliplatin groups. From T1 to T3, a small number of patients in the Cisplatin group exhibited elevated creatinine levels (up to 16.7% at T2), while none in the Carboplatin/Oxaliplatin group showed abnormal values. However, these between-group differences were not statistically significant (*p* > 0.05). 

At time point T3, eGFR was significantly lower in the Cisplatin group (Mean Rank = 17.63) compared to the Carboplatin + Oxaliplatin group (Mean Rank = 27.53), U = 123.00, Z = −2.57, *p* = 0.010. No statistically significant differences in uACR values were observed between the Cisplatin and Carboplatin + Oxaliplatin groups at any time point: T0 (U = 158.50, *Z* = −1.79, *p* = 0.073), T1 (U = 179.50, *Z* = −1.22, *p* = 0.223), T2 (U = 193.00, *Z* = −0.87, *p* = 0.385), and T3 (U = 191.00, *Z* = −0.92, *p* = 0.360). 

For Cystatin C, no significant group differences were detected at T0 through T2. At T3, however, the Cisplatin group showed significantly higher Cystatin C levels than the Carboplatin/Oxaliplatin group (U = 113.50, Z = −2.80, *p* = 0.005). 

uKIM-1 levels did not significantly differ between the cisplatin and carboplatin/oxaliplatin groups at baseline (t0), U = 166.00, Z = −1.52, *p* = 0.129. However, at all subsequent time points, significantly higher uKIM-1 levels were observed in the cisplatin group at t1 (U = 110.50, Z = −2.91, *p* = 0.004), t2 (U = 121.50, Z = −2.67, *p* = 0.007), t3 (U = 122.00, Z = −2.63, *p* = 0.009). These findings indicate a consistent and statistically significant elevation in uKIM-1 among patients receiving cisplatin from the first chemotherapy cycle onward. uClusterin levels did not significantly differ between the cisplatin and carboplatin/oxaliplatin groups at any time point (*p* > 0.05). uNephrin levels were significantly higher in the cisplatin group compared to the carboplatin/oxaliplatin group at t2 (U = 124.50, Z = −2.55, *p* = 0.011). No significant differences were observed at other time points (*p* > 0.05) (Table 12).

### 4.5. Nephrotoxicity Assessment

Nephrotoxicity occurred in 58.1% (*n* = 25) of the patients (a loss of GFR greater than 10 mL/min/1.73 m^2^ between month 0 and month 6), while 41.9% (*n* = 18) either experienced no decline (*n* = 9) or a decline of up to 10 mL/min (*n* = 9). For comparison, when using the criterion of nephrotoxicity as reaching a GFR of 60 mL/min, 23.3% (*n* = 10) met this threshold.

The change in eGFR from baseline to the 6-month follow-up was, on average, negative (Mean = −14.19, SD = 16.40, *n* = 43). The patients in the cisplatin group experienced a significantly greater decline in eGFR at 6 months (Mean = −21.8 mL/min) compared to those in the carboplatin/oxaliplatin group (Mean = −7.27 mL/min) (*p* < 0.001) at 6 months. A decline (>10 mL/min) occurred in 83.3% of the cisplatin group vs. 26.3% of the carbo/oxaliplatin group (χ^2^(1) = 14.17, *p* < 0.001). Among the patients with a sustained decline in eGFR of more than 10 mL/min/1.73 m^2^ at 6 months (*n* = 25), the mean eGFR reduction from baseline was −25.04 ± 11.60 mL/min/1.73 m^2^ (range: −51.00 to −11.00).

Friedman tests were conducted to assess within-group differences in renal biomarkers across four time points (T0–T3) among patients categorized by eGFR decline status at 6 months. In the group with an eGFR decline greater than 10 mL/min/1.73 m^2^, significant differences were observed over time for serum creatinine, χ^2^(3) = 14.57, *p* = 0.002; cystatin C, χ^2^(3) = 26.90, *p* < 0.001; uACR, χ^2^(3) = 10.98, *p* = 0.012; uKIM-1, χ^2^(3) = 14.52, *p* = 0.002; uNephrin, χ^2^(3) = 9.79, *p* = 0.020; and uClusterin, χ^2^(3) = 16.13, *p* = 0.001. Cystatin C levels increased significantly across all post-treatment timepoints compared to baseline (T0–T1/T2/T3; adjusted *p* ≤ 0.001). Urinary KIM-1 levels showed a significant rise at T3 (*p* = 0.003), while uClusterin levels significantly increased from T0 to T2 and T3 (adjusted *p* = 0.013 and 0.003, respectively). Post hoc comparisons for uACR and uNephrin did not reach significance after correction for multiple testing.

In contrast, for patients without a eGFR decline (≤10 mL/min/1.73 m^2^), only uACR showed a significant change over time, χ^2^(3) = 23.00, *p* < 0.001. No significant longitudinal variation was observed for creatinine, cystatin C, uKIM-1, uNephrin, or uClusterin in this group (Table 13).

## 5. Discussion

### 5.1. Nephrotoxicity—Nephrotoxicity in Platinum-Based Therapy

Platinum-based drugs are among the most commonly used antineoplastic therapies, effective against many types of neoplastic diseases. Unfortunately, these drugs exhibit significant toxicity, including nephrotoxicity, which can sometimes limit their use. Nephrotoxicity is a broad term encompassing various types of kidney damage disorders, such as acute kidney injury (AKI), electrolyte abnormalities, isolated proteinuria, and chronic kidney disease (CKD) [22]. 

Various studies apply diverse criteria for nephrotoxicity, making interpretation of the results challenging. In many studies on nephrotoxicity, an eGFR of less than 60 mL/min/1.73 m^2^ is considered a threshold for kidney impairment [23,24]. We acknowledge that adopting a static threshold of 60 mL/min/1.73 m^2^ would be useful if all patients starting antineoplastic drug therapy have a normal eGFR (>90 mL/min/1.73 m^2^) prior to chemotherapy. However, a large study on nearly 5000 oncology patients showed that 50–60% of them had abnormal kidney function, meaning they had an eGFR below 90 mL/min/1.73 m^2^ [5]. When using a static threshold of 60 mL/min/1.73 m^2^, oncology patients who already have a reduced eGFR can easily reach values below 60 mL/min/1.73 m^2^. Therefore, we believe that a dynamic criterion, a decrease in eGFR of more than 10 mL/min/1.73 m^2^ (double the KDIGO criteria for rapid decline in CKD patients), more accurately reflects the development of early nephrotoxicity [25].

Isiiko et al. investigated nephrotoxicity in 206 adult cancer patients who received various chemotherapeutic regimens and reported a 35.9% prevalence of nephrotoxicity among adult cancer patients [26]. In a study focused on platinum-based therapy, of patients receiving combined treatment with docetaxel, cisplatin, and 5-fluorouracil, nephrotoxicity developed in 20 out of 41 patients (48.8%) [27]. In another study involving cancer patients who received at least four cycles of cisplatin chemotherapy, the prevalence of nephrotoxicity caused by cisplatin was reported to be 34.1% [28]. The renal toxicity of carboplatin is around 19%, while nephrotoxicity with oxaliplatin is below 5% [29,30]. In our study, nephrotoxicity, classified as a decline in eGFR of >10 mL/min/1.73 m^2^ at the sixth month after initiation of platinum-based therapy, is 54.3%, (with a startling decline of eGFR of 25 mL/min/1.73 m^2^), with 80% of cases attributable to cisplatin-based therapy. The average decline in the eGFR over a 6-month period in patients on platinum-based regimens is 14.1 mL/min/1.73 m^2^, while in patients receiving high-risk nephrotoxic platinum-based medications, the loss is 21.8 mL/min/1.73 m^2^. Our results confirm a significantly greater risk of nephrotoxicity with cisplatin therapy compared to carboplatin or oxaliplatin. We found that a markedly reduced eGFR was evident in patients treated with cisplatin compared to other less nephrotoxic platinum-based drugs as early as after the third cycle of chemotherapy.

According to an eGFR threshold of 60 mL/min/1.73 m^2^, we found that 10 of the patients, representing 23.3% of the cohort, developed nephrotoxicity. Comparing the two criteria, we report a higher percentage of nephrotoxicity diagnoses when using a threshold of >10 mL/min/1.73 m^2^, which helps identify the presence of potentially hidden nephrotoxicity not previously documented.

### 5.2. Renal Biomarkers in Cancer Patients

Cancer patients represent a unique cohort compared to healthy subjects due to several distinct characteristics related to their disease, treatment, and health outcomes. The values of biomarkers used to assess kidney function in patients with oncological diseases before starting chemotherapy can be significantly altered compared to those in the healthy population. For example, when compared with a reference population, cystatin C concentrations were significantly higher in oncology patients both prior to commencing chemotherapy and during cycles of treatment [31]. Our patient cohort also exhibited increased mean cystatin C values prior chemotherapy, with nearly 75% of cancer patients having cystatin C values above the upper reference range boundary.

Notably, we report that the mean values of uKIM-1 in all 43 oncology patients were significantly increased compared to the healthy volunteers. Until now, only non-significantly elevated KIM-1 values in cancer patients have been observed prior to therapy initiation compared to healthy individuals [32]. Higher values have been recorded in patients with kidney carcinoma, but such patients were not included in our study [33]. Statistically elevated KIM-1 levels have been found with advancing age and in patients with hypertension [34,35]. Accordingly, we found that the patients in our study are statistically significantly older, and most of them have hypertension.

### 5.3. Renal Biomarkers for Early Detection of Platinum-Induced Nephrotoxicity

In general, drug-induced nephrotoxicity represents a major challenge due to the risk of acute and chronic kidney damage. Early recognition, significantly advanced by new urinary and blood biomarkers not used in traditional tests, is essential for effective management and prevention of permanent damage [36,37]. In 2008, the FDA and European Medicines Agency approved seven new biomarkers used for nephrotoxicity detection that may influence clinical decision-making alongside traditional markers: KIM-1, albumin, B2-microglobulin, cystatin C, total protein, clusterin, and trefoil factor-3 [15].

The values of several biomarkers used to evaluate kidney function in cancer patients have shown promising results in improving diagnosis, prognosis, and risk stratification for those with renal diseases.

A study involving 70 patients with various oncological diseases treated with cisplatin evaluated the efficacy of serum cystatin C for assessing impaired kidney function. The serum cystatin C concentration was established to have better sensitivity, specificity, and positive predictive value compared to creatinine in the detection of early stages of renal dysfunction in cancer patients under treatment with cisplatin. In addition, the authors demonstrated that values above 1.28 mg/L predict a decrease in creatinine clearance to below 78 mL/min, with 77% sensitivity and 95% specificity [38]. In patients with eGFR decline >10 mL/min, we found a statistically significant increase in the mean serum cystatin C values with each chemotherapy cycle compared to baseline values. 

In another study involving 124 patients with oncological diseases, Li et al. monitored the changes in serum cystatin C, serum creatinine, urea, and creatinine clearance before the first chemotherapy cycle, after two cycles of chemotherapy, and after four cycles of chemotherapy. Cystatin C continued to increase with prolonged chemotherapy duration. A statistically significant increase was reported after the fourth cycle only in the 71 patients treated with platinum-based (cisplatin or carboplatin) therapy [39]. In our study, we also recorded an increase in the mean values of serum cystatin C after each subsequent chemotherapy cycle, with this trend being evident exclusively in patients treated with cisplatin. A significant 1.4-fold increase was noted as early as after the first chemotherapy cycle. 

Another promising biomarker for the early detection and monitoring of nephrotoxicity in oncology patients is uKIM-1. uKIM-1 concentrations may predict cisplatin-induced AKI in its early stages, with an 87.5% sensitivity and 93.3% specificity [40].

Additionally, George et al. sought to characterise the time-dependent urinary excretion of KIM-1, alongside other novel biomarkers, during two different chemotherapy cycles in 27 patients with solid tumours who were authorised to receive treatment with the anticancer drug cisplatin. In response to the initial cycle, the urinary KIM-1 concentrations increased from baseline and remained elevated through a subsequent cycle of cisplatin chemotherapy [41]. In our study, we also found that the uKIM-1 values increased as early as the first day after the administration of platinum-based agents. Once elevated, the values remained high after each subsequent cycle. These trends were observed regardless of whether the patients were treated with a highly nephrotoxic platinum-based drug or with a moderately toxic and/or low-toxic platinum-based drug. However, the average biomarker values in the patients treated with cisplatin throughout all cycles were statistically elevated (by nearly double) compared to those in the carboplatin/oxaliplatin group. A statistically significant change over time was observed only in the patients treated with cisplatin after the third cycle. Similarly, a statistically significant change in uKIM-1 values after the third cycle was observed in patients with developed nephrotoxicity.

Urinary nephrin also seems to be a promising biomarker for the early diagnosis of kidney injury. The pooled sensitivity of urinary nephrin for detecting glomerular injury was 0.86 (95% CI 0.83–0.89), and the pooled specificity was 0.73 (95% CI 0.70–0.76) [21]. Nephrin has been studied for the assessment of nephrotoxicity associated with various oncological drugs. Most research has focused on anti-VEGF anticancer drugs, revealing that increased urinary nephrin levels at 1 week of treatment predicted the development of nephrotoxicity [13]. Few studies have been conducted on uNephrin as a marker for early nephrotoxicity caused by platinum-based drugs, and most have used animal models. In one study, rats received large intraperitoneal doses of cisplatin. A significant elevation in serum creatinine and urea levels was observed in the cisplatin-treated rats sacrificed at days 5 and 7. The uNephrin level was significantly elevated at day 3 after cisplatin treatment and continued to increase at days 5 and 7. Similar results were reported for uKIM-1 and other novel biomarkers, which were significantly higher on day 3. In the same experiment, the serum Cys C level was significantly elevated only by day 5 and continued to increase through to day 7, when other conventional biomarker levels had also increased [42]. In our study, we observed a statistically significant rise in the mean urinary nephrin levels following the second treatment cycle with cisplatin. However, the findings for patients who developed nephrotoxicity remain unclear. One possible reason for the differing outcomes between the animal and human models may be the variations in the cisplatin dosing protocols used. Cisplatin treatment in patients is often delivered in cycles at 1- or 3-week intervals, comprising either a single high dosage or numerous lower daily doses of cisplatin. In mice or rats, nephrotoxicity is mostly induced by a single cisplatin administration. Repeated cisplatin protocols for nephrotoxicity are extremely rare [43]. In studies on rats, cisplatin has been administered at high sub-lethal doses, while human studies, including ours, utilise standard therapeutic doses.

From the obtained results, we can conclude that the results for uNephrin are inconclusive. This biomarker is probably not suitable for self-conducted assessment of nephrotoxicity in platinum-based regimens. Further extensive research involving human subjects is required to precisely evaluate the effectiveness of this biomarker in detecting cisplatin-related kidney damage. 

Finally, another biomarker approved by the FDA for the early detection of drug-induced nephrotoxicity is urinary clusterin. This biomarker has been studied in both animals and humans. Studies involving cisplatin address multiple forms of renal injury, primarily for the detection of AKI. In preclinical rat studies, as early as 1 day after cisplatin treatment, changes in urinary clusterin indicated the onset of proximal tubular injury in the absence of functional effects. In contrast, serum creatinine and blood urea nitrogen levels were elevated only after 5 days. The authors conclude that tissue Kim-1 and urinary clusterin were the most sensitive biomarkers for detecting cisplatin-induced AKI [44].

Waikar and colleagues investigated six biomarkers, including uClusterin and uKIM-1, in patients treated with intrathoracic cisplatin for malignant pleural mesothelioma. Among those who developed AKI, the increase in urinary clusterin from baseline was statistically significant—2.2-fold compared to healthy controls. The medically significant thresholds for differentiating mesothelioma patients who developed AKI vs. those who did not develop AKI were 5.1-fold for urinary clusterin [45]. Another study proves that certain urinary biomarkers might be particularly sensitive for detecting cisplatin-induced subclinical AKI. By 10 days, uClusterin (1.9-fold) was significantly increased [38]. The peak platinum urinary concentrations at day 10 correlated with the urinary levels of clusterin, alongside KIM-1 and cystatin C [46].

In our study we found a 2.7-fold increase after the first cycle for patients on cisplatin-based treatments. Subsequently, there was a consistent upward trend in the mean uClusterin concentrations after each chemotherapy cycle, with a significant increase after the third cycle (21.1-fold). For patients at moderate/low risk of nephrotoxicity, the biomarker levels increased after the second and third cycles, but without any distinct pattern, and these rises were not statistically significant. Moreover, these values were much lower than those registered in the high-risk cohort, with a significant difference observed between groups after the third cycle. Among the patients who developed nephrotoxicity, the urinary clustering values rose significantly as early as after the second chemotherapy cycle. 

In our research, the performance of conventional biomarkers for the early assessment of nephrotoxicity was controversial. While the mean serum creatinine levels in patients with a high risk of nephrotoxicity rose over the course of the study, only four patients exhibited serum creatinine measurements exceeding the upper reference limit at any point. Trends and fluctuations in creatinine over time are important, but the absolute elevation beyond the upper range serves as a practical, actionable threshold for identifying kidney issues in clinical settings.

In terms of uACR, we identified five patients with levels exceeding the upper reference range following the first cycle of platinum-based therapy, with this number rising over time. After the third cycle, there was a 2.3-fold increase, along with a significant rise in average values among patients receiving cisplatin. Other studies have shown similar results, with cisplatin treatment causing a 2-fold rise by day 10 after administration [47]. Furthermore, we observed a significant increase in uACR even in patients with low to moderate risk of nephrotoxicity, including those without obvious clinical signs of kidney damage.

## 6. Conclusions

Our study’s findings suggest that uKIM-1, uClusterin, and cystatin C are valuable biomarkers for the early identification of cisplatin-induced nephrotoxicity. However, further extensive studies involving larger cohort of patients are needed to establish the clinical role of these biomarkers for early detection of drug-induced nephrotoxicity in oncology patients, identify suitable threshold values, and possibly incorporate them alongside standard clinical tests in everyday practice.

### Limitations

Our study has several limitations. Firstly, we defined nephrotoxicity as a decline in eGFR greater than 10 mL/min/1.73 m^2^ at the sixth month following treatment initiation, whereas other studies employed an eGFR of less than 60 mL/min/1.73 m^2^ as their defining criterion. Secondly, our evaluation of nephrotoxicity was based on a single criterion (eGFR decline) rather than a comprehensive set of markers. Additionally, the novel biomarkers (uKIM-1, uClusterin, uNephrin) were not adjusted for urinary creatinine levels. Another limitation is the demographic heterogeneity between groups, especially in terms of sex distribution. In oncology, treatment assignments are clinically driven, making demographic matching impractical. Finally, given the small sample size, statistical adjustment for confounders like age and sex was also not feasible. As a pilot, hypothesis-generating study, no formal power calculation was performed. The findings are exploratory and intended to guide future larger studies with appropriate statistical adjustment.

## Figures and Tables

**Table 1:** Demographic and clinical characteristics of participants.

Characteristic	Cisplatin (*n* = 24)	Carboplatin/Oxaliplatin (*n* = 19)	Control (*n* = 23)
Age (Mean ± SD)			
male	58.9 ± 13.5	60.8 ± 9.2	34.4 ± 10.2
female	64.0 ± 8.3	62.5 ± 11.1	33.0 ± 8.5
Sex	% (*n*)		
male	79.2% (19)	31.6% (6)	39.1% (9)
female	20.8% (5)	68.4% (13)	60.9% (14)
ECOG PS			
≥2	37.5% (9)	0.0% (0)	—
<2	62.5% (15)	100% (19)	—
Comorbidities			
Hypertension			
Yes	66.7% (16)	63.2% (12)	—
No	33.3% (8)	36.8% (7)	—
Anemia			
Yes	45.8% (11)	42.1% (8)	—
No	54.2% (13)	57.9% (11)	—
Ischemic Heart Disease			
Yes	29.2% (7)	5.3% (1)	—
No	70.8% (17)	94.7% (18)	—
Diabetes			
Yes	12.5% (3)	15.8% (3)	—
No	87.5% (21)	84.2% (16)	—
Chronic obstructive pulmonary disease			
Yes	12.5% (3)	15.8% (3)	—
No	87.5% (21)	84.2% (16)	—

**Table 2:** Distribution of Cancers patients by body location/system.

Body Location/System	Number of Patients (%)
Gastrointestinal	16 (37.2%)
Genitourinary	9 (20.9%)
Respiratory/Thoracic	8 (18.6%)
Gynecologic	5 (11.6%)
Head and Neck	3 (7.0%)
Non site specific	2 (4.7%)

**Table 3:** Baseline biomarker (t0) in Cisplatin, Carboplatin/Oxaliplatin and control groups.

Biomarker (Reference Range)	Cisplatin Group, (*n* = 24)	Carboplatin/ Oxaliplatin, (*n* = 19)	Control Group, (*n* = 23)	*p*	Pairwise Significance (2-Tailed)
Creatinine, µmol/L Male: 74–134 Female: 44–96	80.0 (66.5–95.5) 84.0 (77.0–102.0) 67.0 (58.0–80.0)	68.0 (65.0–77.0) 84.5 (77.0–88.0) 67.0 (65.0–68.0))	–	0.058	-
eGFR (mL/min/1.73 m^2^)	90.5 (71.5–100.5)	87.0 (84.0–94.0)	–	0.807	-
Cystatin C, mg/L (0.51–1.09)	1.42 (1.02–1.88)	1.51 (1.05–1.87)	–	0.854	-
uACR, mg/mmol (0.0–3.0)	3.30 (1.45–3.00)	1.10 (0.60–3.00)		0.730	
uKIM-1 (pg/mL)	672.5 (335.3–1393.1)	511.5 (108.6–1073.1)	198.1 (65.1–417.1)	<0.001	Cis vs. Control, *p* < 0.001; Carbo/Oxa vs. Control, *p* = 0.024; Cis vs. Carbo/Oxa, *p* = 0.130;
uNephrin (ng/mL)	0.20 (0.00–0.30)	0.00 (0.00–0.30)	0.10 (0.00–0.30)	0.710	NS
uClusterin (μg/mL)	1.10 (0.35–2.00)	0.70 (0.30–3.70)	1.40 (0.30–2.60)	0.985	NS

Data are presented as median (interquartile range). Comparisons between groups were performed using the Kruskal–Wallis test; pairwise comparisons were conducted with the Mann–Whitney U test. NS = not significant.

**Table 4:** Changes in Renal Biomarkers in the Cisplatin Group (Mean ± SD).

Biomarker	T0	T1	T2	T3
Creatinine (µmol/L)	84.00 ± 22.49	91.08 ± 24.24	93.71 ± 23.12	97.29 ± 30.06
eGFR (mL/min/1.73 m^2^)	86.71 ± 16.97	81.25 ± 18.14	79.29 ± 19.85	77.71 ± 20.22
Cystatin C (mg/L)	1.46 ± 0.60	1.96 ± 0.86	2.13 ± 0.88	2.40 ± 0.92
uACR (mg/mmol)	2.30 ± 1.07	2.32 ± 1.04	2.60 ± 1.28	5.54 ± 10.95
uKIM-1 (pg/mL)	900.05 ± 672.13	1422.48 ± 633.64	1488.21 ± 703.13	1459.78 ± 712.37
uNephrin (ng/mL)	0.26 ± 0.47	0.75 ± 1.28	1.00 ± 1.31	0.79 ± 1.04
uClusterin (μg/mL)	1.96 ± 2.68	6.73 ± 9.42	9.01 ± 14.99	15.95 ± 19.91

**Table 5:** Friedman Test and Post Hoc Wilcoxon Comparisons for Renal Biomarkers in the Cisplatin Group (*n* = 24).

Biomarker	*n*	Mean Rank				χ^2^	df	*p*	Significant Pairwise Comparisons (Wilcoxon z, *p*)
		t0	t1	t2	t3				
uACR	24	2.08	2.19	2.56	3.17	15.16	3	0.002	t0–t3: z = −2.907, *p* = 0.022
Cystatin C	24	1.42	2.5	2.79	3.29	27.15	3	<0.001	t0–t1: z = −2.91, *p* = 0.022 t0–t2: z = −3.69, *p* = 0.001 t0–t3: z = −5.03, *p* < 0.001
uKIM-1	24	1.77	2.71	2.65	2.88	12.67	3	0.005	t0–t3: z = −2.96, *p* = 0.018
uNephrin	24	1.73	2.52	3.0	2.75	14.19	3	0.003	t0–t2: z = −3.41, *p* = 0.004 t0–t3: z = −2.74, *p* = 0.037
uClusterin	24	1.77	2.35	2.69	3.19	15.67	3	0.001	t0–t3: z = −3.80, *p* = 0.001

**Table 6:** Fold Change in Renal Biomarkers Over Time in Cisplatin Group.

Biomarker (Fold Change)	*n*	Mean ± SD
Cystatin C t0–T1	24	1.41 ± 0.42
Cystatin C t0–T2	24	1.63 ± 0.76
Cystatin C t0–T3	24	1.95 ± 1.38
uKIM-1 t0–T1	24	3.51 ± 4.17
uKIM-1 t0–T2	24	3.79 ± 5.08
uKIM-1 t0–T3	24	3.78 ± 4.99
uClusterin t0–T1	24	9.35 ± 18.2
uClusterin t0–T2	24	10.42 ± 24.64
uClusterin t0–T3	24	21.6 ± 38.24
uNephrin t0–T1	24	3.46 ± 4.3
uNephrin t0–T2	24	4.94 ± 5.41
uNephrin t0–T3	24	3.83 ± 4.17

**Table 7:** Friedman Test for Fold Change in Renal Biomarkers Across Timepoints in Cisplatin Group.

Biomarker	*n*	Mean Rank			χ^2^	df	*p*
		T0–T1	T0–T2	T0–T3			
Cystatin C	24	1.63	1.96	2.42	7.583	2	0.023
uKIM-1	24	2.02	1.85	2.13	1.229	2	0.541
uNephrin	24	1.79	2.23	1.98	2.494	2	0.287
uClusterin	24	1.69	1.94	2.38	5.936	2	0.051

**Table 8:** Changes in Renal Biomarkers in the Carboplatin/Oxaliplatin group (Mean ± SD).

Biomarker	T0	T1	T2	T3
Creatinine (µmol/L)	70.95 ± 10.81	72.58 ± 8.75	71.53 ± 10.54	68.79 ± 9.11
Cystatin C (mg/L)	1.46 ± 0.63	1.68 ± 0.85	1.65 ± 0.88	1.65 ± 0.50
eGFR (mL/min/1.73 m^2^)	90.11 ± 8.83	88.00 ± 11.66	89.05 ± 11.02	92.58 ± 9.62
uACR (mg/mmol)	1.58 ± 1.19	1.87 ± 1.12	2.13 ± 1.22	3.60 ± 3.79
uKIM-1 (pg/mL)	645.43 ± 626.96	796.64 ± 529.23	838.07 ± 676.84	844.79 ± 582.86
uNephrin (ng/mL)	0.28 ± 0.59	0.37 ± 0.60	0.29 ± 0.40	0.57 ± 1.20
uClusterin (μg/mL)	2.64 ± 3.73	2.07 ± 2.15	7.08 ± 15.44	4.93 ± 6.22

**Table 9:** Friedman Test and Post Hoc Wilcoxon Comparisons for Renal Biomarkers in Carboplatin/Oxaliplatin group.

Biomarker	*n*	χ^2^	df	*p*
Creatinine (within reference range)	19	-	-	N/A
eGFR	19	3.33	3	0.344
uACR	19	16.07	3	0.001
Cystatin C	19	1.80	3	0.615
uKIM-1	19	2.90	3	0.407
uNephrin	19	1.97	3	0.580
uClusterin	19	2.10	3	0.552

**Table 10:** Fold Change in Renal Biomarkers Over Time in Carboplatin/Oxaliplatin Group.

Biomarker (Fold Change)	*n*	Mean ± SD
Cystatin C t0–T1	19	1.39 ± 1.05
Cystatin C t0–T2	19	1.38 ± 1.26
Cystatin C t0–T3	19	1.27 ± 0.51
uKIM-1 t0–T1	19	3.13 ± 3.6
uKIM-1 t0–T2	19	3.56 ± 5.62
uKIM-1 t0–T3	19	8.57 ± 21.82
uClusterin t0–T1	19	2.74 ± 3.84
uClusterin t0–T2	19	13.16 ± 38.99
uClusterin t0–T3	19	11.32 ± 24.82
uNephrin t0–T1	19	2.54 ± 4.61
uNephrin t0–T2	19	1.93 ± 2.1
uNephrin t0–T3	19	2.43 ± 3.09

**Table 11:** Friedman Test for Fold Change in Renal Biomarkers Across Timepoints Carboplatin/Oxaliplatin Group.

Biomarker (Fold Change)	*n*	Mean Rank			*χ^2^*	df	*p*
		T1	T2	T3			
Cystatin C	19	2.05	1.84	2.11	0.737	2	0.069
uKIM-1	19	1.87	2.08	2.05	0.535	2	0.765
uClusterin	19	1.68	2.24	2.08	3.12	2	0.210
uNephrin	19	1.87	2.05	2.08	0.826	2	0.662

**Table 12:** Mann–Whitney U Test Results for Cystatin C and eGFR between Cisplatin and Carboplatin/Oxaliplatin groups.

Biomarker	Time Point	Cisplatin Mean Rank	Carbo/Oxali Mean Rank	U	Z	*p*-Value
eGFR	T3	17.63	27.53	123.0	−2.57	0.01
Cystatin C	T3	26.77	15.97	113.5	−2.8	0.005
uKIM-1	T1	26.9	15.82	110.5	−2.905	0.004
	T2	26.44	16.39	121.5	−2.674	0.007
	T3	26.42	16.42	122.0	−2.629	0.009
uNephrin	T2	26.31	16.55	124.5	−2.553	0.011

**Table 13:** Biomarker Median (25th–75th Percentile) Values by Group and Time Point.

Biomarker	Group	Median (25th–75th Percentile)						
		t0	t1	t2	t3	χ^2^	df	*p*-Value
Creatinine	Decline > 10 mL/min	78.0 (63.5–84.5)	81.0 (65.5–100.0)	79.0 (70.0–103.0)	87.0 (71.0–101.5)	14.573	3	0.002
	No Decline or decline < 10 mL/min	73.0 (65.8–88.8)	78.0 (67.8–84.5)	73.0 (68.8–86.3)	70.5 (64.5–82.3)	3.157	3	0.368
Cystatin C	Decline > 10 mL/min	1.29 (0.87–1.83)	1.96 (1.30–2.72)	2.08 (1.51–2.72)	2.25 (1.56–2.85)	26.904	3	<0.001
	No Decline or decline < 10 mL/min	1.63 (1.22–1.87)	1.48 (0.87–1.87)	1.27 (1.18–2.17)	1.75 (1.34–2.24)	4.733	3	0.192
uACR	Decline > 10 mL/min	3.00 (1.15–3.00)	3.00 (1.35–3.00)	2.80 (1.60–3.00)	3.00 (2.10–4.35)	10.976	3	0.012
	No Decline or decline < 10 mL/min	1.15 (0.60–3.00)	1.80 (0.75–3.00)	1.90 (1.48–3.00)	3.00 (1.55–3.55)	23.0	3	<0.001
uKIM-1	Decline > 10 mL/min	596.5 (248.8–1126.8)	1249.1 (600.5–2000.0)	1521.1 (592.5–2000.0)	1649.1 (752.5–2000.0)	14.516	3	0.002
	No Decline or decline < 10 mL/min	554.0 (164.9–1880.8)	965.5 (559.5–1364.8)	704.5 (266.3–2000.0)	647.6 (370.3–1634.8)	3.0	3	0.392
uNephrin	Decline > 10 mL/min	0.00 (0.00–0.30)	0.10 (0.00–0.75)	0.30 (0.10–1.00)	0.40 (0.05–0.95)	9.786	3	0.02
	No Decline or decline < 10 mL/min	0.10 (0.00–0.33)	0.15 (0.00–0.83)	0.40 (0.00–0.98)	0.10 (0.00–0.70)	6.605	3	0.086
uClusterin	Decline > 10 mL/min	0.90 (0.30–2.25)	1.59 (0.50–8.70)	4.20 (1.15–6.45)	5.60 (0.75–25.5)	16.131	3	0.001
	No Decline or decline < 10 mL/min	0.85 (0.38–3.55)	2.20 (0.50–4.48)	2.30 (0.25–6.68)	3.20 (0.85–9.18)	1.034	3	0.793

## Data Availability

The raw data supporting the conclusions of this article will be made available by the authors on request.

## Supplementary Materials

The following supporting information can be downloaded at: https://www.mdpi.com/article/10.3390/life15091432/s1, Table S1: Distribution of patients in the high-risk group for nephrotoxicity according to the chemotherapy regimen; Table S2: Distribution of patients in the low-to-moderate risk group for nephrotoxicity according to the chemotherapy regimen.
