# Supplementary material for: Biomarkers for Early Detection of Cisplatin-Induced Nephrotoxicity
Source: Life (Basel). 2025 Sep 12;15(9):1432. doi: 10.3390/life15091432 (PMC12471489; doi:10.3390/life15091432)
Supplement: Supplementary file 1 [file life-15-01432-s001.zip › life-3837601-supplementary.pdf]

Supplementary Table S1. Distribution of patients in the high-risk group for nephrotoxicity according to the chemotherapy regimen.

**Antineoplastic regimens**	**Number of patients (%)**
Cisplatin/Gemcitabine	9 (37.5%)
Cisplatin/Taxane +/- 5-fluorouracil	5 (20.8%)
Cisplatin/Etoposide +/- Bleomycin	5 (20.8%)
Cisplatin/Epirubicin/5-fluorouracil	2 (8.3%)
Cisplatin/Capecitabin	2 (8.3%)
Cisplatin/Vinorelbin	1 (4.2%)

Supplementary Table S2. Distribution of patients in the low-to-moderate risk group for nephrotoxicity according to the chemotherapy regimen.

**Antineoplastic regimens**	**Number of patients (%)**
Carboplatin/Taxane	7 (36.9%)
FOLFOX-4	5 (26.3%)
FOLFIRINOX	2 (10.5%)
Oxaliplatin/Gemcitabine	2 (10.5%)
Oxaliplatin/Capecitabin	2 (10.5%)
Oxalipatin/Irinotecan	1 (5.3%)
